# Editorial: Sleep disorders, hypertension and cardiovascular diseases

**DOI:** 10.3389/fcvm.2022.1110487

**Published:** 2022-12-19

**Authors:** Valeria Bisogni, Giuseppe Maiolino, Martino F. Pengo

**Affiliations:** ^1^Unit of Internal Medicine, Terni University Hospital, Terni, Italy; ^2^Clinica Medica 3, Department of Medicine – DIMED, University of Padua, Padua, Italy; ^3^Department of Cardiovascular, Neural and Metabolic Sciences, IRCCS Istituto Auxologico Italiano, Milan, Italy; ^4^Department of Medicine and Surgery, University of Milano-Bicocca, Milan, Italy

**Keywords:** sleep-related disorders, obstructive sleep apnoea, restless legs syndrome, cardiovascular diseases, arterial hypertension

Clinical studies on ambulatory blood pressure (BP) have shown that increases in BP values, particularly at nighttime, are associated with cardiovascular (CV) morbidity and mortality (1, 2). Accordingly, sleep-related disorders that induce rises in nighttime BP can contribute to further increase CV risk (3–5). The underlying mechanisms supporting this association include sympathetic nervous system activation, oxidative stress, systemic inflammation, endothelial dysfunction, and renin-angiotensin-aldosterone activation. Most of them have been demonstrated in both animal and human studies particularly for sleep-disordered breathing (SDB) such as obstructive sleep apnoea (OSA) (6).

In this Research Topic, we have included several articles trying to further elucidate the relationship between sleep disorders (particularly OSA), systemic arterial hypertension, and CV disease. OSA is indeed the most common sleep-related disorder and is associated with hypertension and CV comorbidities. It affects 10% of adult females and 20% of males with prevalence that can rise up to 40–90% in specific subgroups like patients with severe obesity (7).

In this regard, in the study by Bock et al. it was hypothesized that central adiposity measured by the waist-to-hip ratio (WHR), which appears to be more closely related to the presence of OSA as compared to the body mass index (BMI) (8), might be superior to BMI when predicting ambulatory overnight oximetry (OXI) results. To this point, they examined clinical and anthropometric factors (BMI, WHR), which predicted abnormal OXI, and the need for subsequent polysomnography in a population of 393 men. The presence of abnormal OXI was high (75%), and the strongest predictor of abnormal OXI was the WHR, followed by BMI, age ≥ 55 yrs, and the presence of snoring. A strong association was observed between WHR and abnormal OXI both in obese and non-obese subjects aged ≥ 55 yrs. An interesting point was raised by the authors: WHR is simple, relatively easy to calculate and interpret with minimal cost instrument capable of predicting CV risk. Accordingly, patients triaged to overnight OXI *via* WHR measurement may avoid lengthy wait times for inpatient polysomnography, and they could initiate a proper treatment sooner.

The treatment of OSA was discussed in the mini review by Arachchige and Steier. They comprehensively described all possible types of OSA treatment available nowadays, including several non-CPAP strategies, such as hypoglossal nerve stimulation and transcutaneous approaches (9). Nevertheless, OSA treatment remains a challenge, and it is necessary to move away from a “one-size-fits-all” strategy and establish a multidisciplinary approach for each patient.

In the second original research by Imanari et al. it was discussed a particular aspect of SBD treatment: adaptative servo-ventilation (ASV). This device can be used in treating SDB in patients with heart failure (HF) and provide information about their residual respiratory events (10). Therefore, the study purpose was to compare, in patients with HF, the apnea-hypopnea index (AHI), determined by the ASV device AutoSet CS (ASC) and with the AHI calculated by polysomnography (PSG). The results are interesting because there was only a modest correlation between the AHI-PSG and AHI-ASC. A possible explanation raised by the authors is related to the presence of central respiratory events during wakefulness or to the attenuation of flow signal amplitude without either arousal or oxygen desaturation, both of which are often observed in patients with HF. According to these findings, when using ASC devices clinicians should consider this discrepancy during the assessment of residual AHI.

Lastly, in the field of OSA treatment and its CV complications, the study protocol by Tavoian et al. has been designed to assess the effects of 24 weeks of high-resistance inspiratory muscle strength training (IMST) on blood pressure (BP) and vascular function. IMST is performed on a portable hand-held device and entails repeated inspiratory efforts against a resistance. It is a simple and time-efficient breathing exercise consistently reported to reduce systolic BP, sympathetic nervous system activity, and endothelial dysfunction in small, selected groups of healthy and high-risk populations at a short-term follow-up (11). Nevertheless, results for OSA patients are still preliminary and require confirmation in a larger group of patients. Based on this evidence, the authors proposed a single-site, double-blind, randomized clinical trial. The primary aim is to observe at least 15-mmHg reduction of systolic BP in middle-aged and older subjects when high-resistance IMST is performed over longer periods (i.e., >6 months). Interestingly, they will try to clarify the underlying mechanisms driving IMST-induced BP reduction, such as vascular endothelial function, aortic stiffness, and nitric oxide and reactive oxygen species production.

Overall, we think that the “*Sleep disorders, hypertension and cardiovascular diseases*” Research Topic met its objectives by making a significant contribution to a number of different areas of this field, even though there are still many aspects that need to be resolved and better understood regarding the association between sleep disorders and cardiovascular diseases. We hope that the readers will find this Research Topic inspiring and helpful in both research and clinical management of OSA.

## Author contributions

All authors listed have made a substantial, direct, and intellectual contribution to the work and approved it for publication.
